# Hidden in the Data: Breast Cancer Patterns, Gaps, and Challenges for Native American Women

**DOI:** 10.1007/s12609-025-00629-7

**Published:** 2026-01-06

**Authors:** Jennifer Erdrich, Hannah E. Woriax, Aaron Thomas, Michelle R. Huyser

**Affiliations:** 1 Department of Surgery, University of Arizona College of Medicine, Turtle Mountain Ojibwe, Tucson, 1501 N. Campbell Ave, AZ 85724 USA; 2 Duke University, Lumbee, Durham, NC USA; 3 University of Arizona, Diné (Navajo), Tucson, AZ USA; 4 Department of Surgery, University of Missouri, Diné (Navajo), Columbia, MO USA

**Keywords:** Breast cancer, American indian, Alaska native, Native american, Cancer disparities

## Abstract

**Purpose of the Review:**

This paper aims to provide a comprehensive overview of breast cancer screening, clinical characteristics, treatment, and survivorship in American Indian/Alaska Native (AI/AN) women.

**Recent Findings:**

Breast cancer in AI/AN women is a complex public health issue marked by disparities in incidence, stage, treatment, and survival that vary regionally. Limited screening mammography, workforce shortages, and underfunded healthcare systems contribute to late diagnoses and poorer outcomes. Social and cultural factors, including historical trauma and medical mistrust, further hinder care. Data on breast cancer topics for AI/AN is limited and often compromised by misclassification and underrepresentation. The Indian Health Service (IHS) plays a crucial but underfunded role in providing care, often insufficient to meet the complex needs of AI/AN women, especially those living in urban areas.

**Summary:**

As Indigenous surgeons working with AI/AN communities, we applied our perspectives to a comprehensive review of the literature. To reduce disparities, research must prioritize Indigenous leadership, respect tribal sovereignty, improve data accuracy, and foster equitable collaborations that center AI/AN priorities.

**Definitions:**

The United States (US) Census Bureau defines American Indian and Alaska Natives (AI/ANs) as persons with origins from the Indigenous peoples of the Americas who maintain tribal affiliation and/or community attachment. Common terms for AI/ANs today include Native, Native American, Indigenous, First Nations, Native Hawaiian, Pacific Islander, among others. The most appropriate phrasing would be to use the specific tribe’s self-designated name in that tribe’s native language when possible. However, for simplicity and the purposes of this manuscript, we will use the term AI/AN.

## Introduction

American Indian and Alaska Native (AI/AN) populations represent over 574 federally recognized tribes across the US, each with its own distinct culture, language, and traditions. Despite their rich heritage and sovereign political status, AI/AN communities face some of the starkest health disparities in the nation. The origins of these disparities lie in the historical legacy of colonization, forced relocation, cultural erasure, and broken federal treaties, which have collectively undermined the social, economic, and physical well-being of Native communities for generations. Today, AI/ANs experience higher rates of poverty, lower access to education and preventive services, and some of the lowest life expectancies of any racial or ethnic group in the US [1]. Nearly 60–70% of AI/ANs live in urban areas—often outside of tribal lands—where access to culturally competent healthcare resources is severely limited [2, 3]. These structural inequities set the stage for disproportionate disease burdens, including a rising incidence of cancer.

In 2024, approximately 310,720 new cases of breast cancer were identified among women in the US. Breast cancer is the second leading cause of cancer death among women with 42,250 women dying from breast cancer in 2024, mostly greater in women ≥ 50 years old [4]. Among AI/AN women, breast cancer is the most diagnosed cancer and leading cause of cancer morbidity and mortality [5, 6]. Although concerted efforts to reduce the overall death rate among many different racial groups from breast cancer have been successful, rates for AI/AN women have remained unchanged [4, 6]. 

While national data may suggest that breast cancer incidence in AI/AN women is slightly lower than in non-Hispanic White (NHW) women, such aggregate figures mask significant regional and tribal variability. Compounding this burden AI/AN women are more likely to be diagnosed at later stages with a higher prevalence of aggressive cancer such as triple-negative breast cancer [5, 7]. AI/AN women are less likely to receive timely mammographic screening, breast-conserving surgery, radiation therapy, chemotherapy, endocrine therapy, and genetic testing [5, 7–9]. This translates to poorer breast cancer survival than NHWs, even after receiving definitive breast cancer therapy [6]. 

Data in this population must be interpreted with caution for a myriad of reasons. AI/ANs represent a heterogenous population split between urban and rural areas throughout the US. Most of the breast cancer information we have about this patient population comes from large population databases or research studies where AI/ANs are often misclassified, merged into other ethnic data groups, or wholly dismissed. Efforts to mitigate these limitations require classification ratios with purchased/referred care (PRC) delivery area counties of the IHS, which largely represent a non-urban AI/AN population and only 34–56% of the overall AI/AN population, resulting in a large loss of AI/AN data [6, 7]. 

Most of what we know about the status of AI/AN health comes from the IHS, which was established in 1955 and is the primary federal agency tasked with fulfilling the US government’s treaty obligation to provide healthcare to AI/AN people [10]. Today the IHS delivers services to 2.8 million AI/ANs, approximately 1/3rd of the AI/AN population, throughout the US [11]. The IHS delivers services through a combination of federally operated hospitals and clinics, tribally operated health systems, and urban Indian health programs. PRC is the method in which the IHS pays outside providers for subspecialty services not provided by the IHS directly. These funds are subject to individuals based on strict eligibility requirements dependent on residency and medical priority [12, 13]. While IHS provides essential primary and preventive care, it is chronically underfunded, receiving approximately $4,000 per user annually, less per capita healthcare spending compared to all other federally funded groups including Medicaid recipients, prisoners, veterans, and military personnel [14, 15]. This funding gap translates into outdated facilities, severe workforce shortages, and limited access to diagnostic and specialty services such as mammography, oncology, and surgical care [16]. Specialized care is often only available through PRC. As a result, many AI/AN women—particularly those living in urban areas or outside of designated service areas—are effectively excluded from receiving cancer diagnostics and treatment through the IHS system. Moreover, even when services are technically available, additional barriers such as transportation, and historical medical mistrust can lead to delays in diagnosis, incomplete treatment, and reduced survivorship [17]. 

Breast cancer care among AI/ANs can be complex and difficult to understand for many reasons. Few papers look specifically at AI/AN breast cancer care in the modern era; most are often outdated. In this paper, we seek to provide a more in-depth overview of AI/ANs breast cancer care including screening, clinical characteristics, treatment, and survivorship.

## Imaging

Timely and accessible imaging is fundamental to the early detection and treatment of breast cancer. Mammography remains the gold standard for screening and has demonstrated mortality reduction through early-stage detection. However, AI/AN women experience significant disparities in accessing mammography, contributing to later-stage diagnoses and poorer breast cancer outcomes [5, 7, 8]. 

Multiple studies have shown that AI/AN women are less likely than NHW women to receive screening mammograms, even when controlling for insurance status, income, and comorbidities [10]. These disparities stem partly from limited access to imaging infrastructure. Many IHS and tribally operated facilities lack on-site mammography services [13]. Patients are often required to travel substantial distances for screening, resulting in decreased uptake and delayed diagnosis [13, 16]. While PRC helps bridge this gap, its underfunding and restrictive eligibility impede radiology access [13, 14]. Facility limitations also play a role in the disparity. A 2023 report by the US Government Accountability Office noted that many federal IHS facilities operate with outdated or insufficient medical imaging equipment, and radiology services are frequently dependent on contracted or telemedicine solutions that may not meet local demand. Workforce shortages further contribute to imaging delays, particularly in regions experiencing high disease burden [16]. 

Cultural and historical factors compound these structural challenges. Mistrust of the medical system, rooted in historical trauma and ongoing experiences of bias, may reduce participation in breast cancer screening [17]. Culturally tailored outreach programs, navigators, and the integration of community health workers have been shown to improve imaging participation and should be considered part of a multifaceted strategy to reduce disparities [17]. In the Southwest, community-based screening initiatives helped raise mammography rates and lower late-stage diagnosis rates compared to other regions [5]. In contrast, AI/AN women in the Northern Plains and Southern Plains are more likely to present with advanced-stage disease, reflecting disparities in imaging access and care delivery [5, 6]. 

Efforts to close the imaging gap for AI/AN women must include federal and tribal investment in mobile mammography units, radiology infrastructure, workforce development, and policy changes that ensure equitable access to diagnostic services through PRC. Without such targeted interventions, imaging disparities will continue to drive inequities in breast cancer outcomes for AI/AN women.

## Cancer Staging

Among AI/AN women with breast cancer, a disproportionate number are diagnosed at a later stage compared to their NHW counterparts—a persistent pattern over decades contributing to the persistent mortality gap [5–7]. Late-stage presentation is strongly associated with lower survival, greater treatment intensity, and higher healthcare costs [8]. 

Data from the American Cancer Society’s special report on cancer in AI/ANs reveal substantial geographic variation in stage at diagnosis. In the Southwest, approximately 57.5% of breast cancers among AI/AN women are diagnosed at local stages, whereas in Alaska, this figure rises to 66.4% [5]. Nationally, however, AI/AN women are less likely to be diagnosed with early-stage breast cancer and more likely to present with regionally or distantly advanced disease [6, 7]. Notably, Kratzer et al. (2023) found that even after controlling for insurance and geographic location, AI/AN women were more likely to be diagnosed with regional or distant stage breast cancer compared to NHWs.

Compounding this trend is the elevated prevalence of aggressive tumor subtypes in AI/AN women. Triple-negative breast cancer (TNBC) is more common in AI/AN women than in NHWs [5, 17]. This subtype often presents at more advanced stages and progresses rapidly, underscoring the importance of early detection. Furthermore, AI/AN women are diagnosed at a younger age—nearly 30% are diagnosed before age 50—intersecting with this elevated TNBC burden and reduced access to age-appropriate screening [4–6]. 

Many AI/AN communities lack proximity to comprehensive cancer centers or diagnostic facilities, resulting in significant geographic and transportation barriers [10]. This contributes to delays in diagnosis, as does limited availability of mammography and biopsy services within IHS and tribally operated facilities, in addition to the described underfunding, workforce shortages, trauma and mistrust that further hinder timely care-seeking behaviors [15, 16]. The impact of these barriers is deeply consequential. Later-stage diagnosis remains one of the primary mediators of survival disparity between AI/AN and NHW women, even after adjustment for treatment and comorbidity [6–8]. Despite national efforts to increase screening rates and stage-appropriate therapy, these initiatives have not translated into significant stage migration in many AI/AN communities.

## Surgery

Women preparing for breast cancer surgery choose between lumpectomy and mastectomy. It is well documented that modern multimodal treatment confers equivalent survival and recurrence between these operations [18, 19]. Lumpectomy has the advantage of less surgical risk, faster recovery, more favorable cosmetics and better patient satisfaction compared to mastectomy [20]. Unfortunately, AI/AN women experience disproportionately greater frequency of mastectomy compared to the general population. A Surveillance, Epidemiology, and End Results (SEER) analysis with IHS linkage data from 2010 to 2015 showed significantly higher percentage of AI/AN women with early stage breast cancer were treated with mastectomy (41% versus 34.4%, *p* < 0.001) [9]. Important regional variations in these data demonstrate wider gaps in the Northern Plains where the percentage of mastectomy for AI women was 48.9% compared to 35.9% for NHW women, and Alaska where 47% of AN women underwent mastectomy compared to 33.3% of NHW women, *p* < 0.001 [9]. A SEER analysis without IHS linkage data spanning 2000–2015 similarly showed greater likelihood of mastectomy for AI/AN (48% AN, 39% AI, 36% NHW) [21]. A study using data from the American College of Surgeons National Surgical Quality Improvement Program (ACS-NSQIP, 2008–2021) found that AI/AN women had the highest percentage of radical mastectomy at 19%, an operation used sparingly if not avoided in contemporary practice [22]. 

Perioperative disparities in outcomes for mastectomy and lumpectomy for AI/AN women also exist. The above ACS-NSQIP study determined that AI/AN women were the most susceptible to any form of perioperative complications with OR = 1.41, *p* < 0.001 [22]. AI/AN women had increased risk of surgical complications (OR = 2.89 and OR = 1.60, respectively) [22]. 

Breast surgical management can be a sensitive topic for anyone, but known threats to AI/AN women’s reproductive freedom can heighten sensitivity. AI/AN women have been the target of unconsented operations with one of the darkest examples being forced sterilization procedures performed on up to 25% of AI/AN reproductive age women from 1962 to 1976 [23]. Contemporary clinicians should be aware of this because it impacts AI/AN women’s trust in surgical counseling and consent. International Indigenous scholars have issued guidelines for consenting Indigenous women for breast cancer operations and offered practical solutions to improving Indigenous women’s access to their preferred breast cancer operation such as telehealth, transportation, housing, community grants, accelerated radiation courses, direct implant reconstruction, Medicaid/Medicare enrollment, and clustering visits with Surgery, Medical Oncology, and Radiation Oncology [24]. 

## Reconstruction

Given greater mastectomy rates, it is important to evaluate patterns of postmastectomy reconstruction (PMR) for AI/AN women. The Women’s Health and Cancer Rights Act (1998) and the Affordable Care Act (2010) are legislative achievements that improved women’s access to breast reconstruction following oncologic surgery [25]. The first of these federal laws requires all insurance companies provide reconstructive services for their members; however, the IHS is not an insurance program, leaving AI/AN women unprotected by this otherwise landmark legislation. The absence of on-site IHS plastic surgery services leaves AI/AN women further vulnerable because they must be referred outside IHS utilizing PRC [7, 22, 26]. 

PMR improves quality of life and is increasingly adopted by NHW women, but a retrospective cohort study utilizing the National Cancer Database (NCDB) found AI/AN women had significantly decreased likelihood of PMR (OR = 0.62, 95% CI 0.56–0.69) [26]. The study found that while PMR increased for AI/AN women over the study period from 13% to 47%, their rates were persistently lower than NHW women whose rates improved 29% to 62% [26]. Some women make the decision for mastectomy and choose contralateral prophylactic mastectomy (CPM), which does not improve survival but can reduce the risk of future contralateral breast cancer and potentially provide better symmetry. Interestingly, the PMR cohort showed AI/AN women more frequently underwent contralateral prophylactic mastectomy (CPM) compared to their NHW counterparts (49% versus 22%, *p* < 0.001) [26]. The AI/AN women who underwent PMR more frequently resided in urban areas, had higher educational levels, and more often had private insurance [26]. A systematic review and meta-analysis on this subject similarly demonstrated that AI/AN women were significantly less likely to undergo reconstruction (pooled OR = 0.47, 95% CI 0.34–0.66) [27]. A qualitative study involving First Nations women in Canada explored their perceptions and barriers to reconstruction. Participants reported significant mistrust in the healthcare system, financial constraints, distance barriers, lack of information, and cultural dissonance that made them want to avoid interactions with a system they perceived as discriminatory and unresponsive to their cultural identity [28]. 

## Radiation

Given the georemote location of many tribal lands and the demanding schedule of radiotherapy, it is not surprising that AI/AN women with breast cancer face barriers to radiation care. A SEER analysis published in 2014 examining data from 1996 to 2005 demonstrated that AI/AN women were less likely than NHW patients to receive radiation for breast cancer [8]. To our knowledge, no updated large cohort study to assess improvement has been done. A literature review in 2017 on radiation access and utilization of AI/AN patients yielded only 3 studies, which is unchanged today. Radiation access, barriers, utilization, and outcomes are notably understudied for AI/AN patients with breast cancer. Very few dedicated studies focus on AI/AN and most conclusions seem to be drawn from outdated literature or literature that has scant AI/AN data embedded in larger cohort studies where AI/AN representation is < 1% of the study.

The Walking Forward program, led by principal investigator and radiation oncologist Dr. Petereit, was established in 2002 with the purpose of addressing cancer disparities for AI residing in South Dakota [29]. Through community based participatory research, patient navigation, and community education, the program has engaged more than 4000 AI patients for studies involving cancer screening and treatment. Impressively, the program has achieved 10% clinical trial participation [30]. In 2023, they published results from a survey investigating AI women’s perceived and real barriers to breast cancer treatment in an effort to expand their inclusion in a patient navigation effort to reduce radiation disparities. Mistrust of the overall health care system was a prominent theme along with privacy concerns and distance to care [29]. 

In a 2022 SEER study on surgical patterns for AI/AN women with breast cancer, there were no overall differences in post-lumpectomy radiation percentages between AI/AN and NHW women [9]. Interestingly, some geographic regions demonstrated AI/AN women having statistically higher percentage of post-lumpectomy radiation for early stage breast cancer compared to NHW women including the Northern Plains (84.3% vs. 77.5%, *p* = 0.04), Alaska (71.4% versus 59.9%, *p* = 0.03) and the Southwest (55.4% vs. 43.6%, *p* = 0.004). Likewise, an NCDB study reported that AI/AN women received post-lumpectomy radiation more frequently than NHW women (30% versus 28%, *p* = 0.01) [26]. In terms of postmastectomy radiation therapy (PMRT), AI/AN women underwent PMRT less frequently than NHW women (31% versus 48%, *p* ≤ 0.001) [26]. In a 2014 study examining partial breast irradiation (PBI), the authors found that AI/AN women used PBI more than twice as often as their urban counterparts [31]. 

An NCDB study published in 2025 analyzed patterns of radiation refusal and found overall 5.09% of women refused recommended radiation. Stratification by race showed that 4.68% of AI/AN women and 6.8% of Native Hawaiian/Pacific Islander women refused radiation, compared to 4.13% of NHW women [32]. Factors associated with refusing radiation for the overall cohort included older age, higher educational attainment, greater distance to treatment facilities, uninsured status, greater comorbidities, and higher clinical stage [32]. A study of Medicare beneficiaries showed that the interval from surgery to radiation was significantly greater for AI/AN women compared to NHW women [33]. A cross sectional survey study from the Texas Cancer Registry examined racial differences in long-term adverse effects from radiation among breast cancer survivors. Based on the BREAST-Q Adverse Effects of Radiation overall score, AI/AN women had the worst BREAST Q scores (−22.2, *p* = 0.01), which were higher than Black patients (−10.8), and Hispanic patients (−7.8), all of whom reported worse changes in skin pigmentation, telangiectasias, dryness, soreness, and irritation compared to NHWs [34]. 

## Chemotherapy

Reports about chemotherapy utilization for AI/AN women are at times conflicting, but a common conclusion among studies describing chemotherapy patterns is AI/AN women with breast cancer are less likely to receive guideline concordant care. In an often cited article by Javid et al., a SEER analysis demonstrated that AI/AN patients were less likely to receive recommended adjuvant chemotherapy for breast cancer than NHW patients (*p* < 0.001), and refusing chemotherapy had an overall significantly lower survival (HR 0.56, 95% CI 0.53–0.59) [8]. Overall, AI/AN breast cancer patients have longer intervals from time of surgery to initiation of adjuvant chemotherapy compared to NHW women, negatively impacting outcomes [35]. A study published in 2024 based on NCDB data from 2004 to 2020 looking at declination of treatment in breast cancer found that their group called “AI/AN or other” were more likely to decline chemotherapy compared to NHW patients (1.13, 95% CI 1.05–1.21), though the compiling of AI/AN into the “other” category is highly problematic for drawing meaningful interpretation [36]. In a SEER study using data from 2000 to 2017, AI/AN women had the lowest proportion of patients receiving HER2-targeted therapy among the patients with Her2/neu positive tumors [37]. Evidence shows AI/AN women are more likely to be diagnosed with TNBC, for which chemotherapy is standard for all patients who can tolerate it, but no research was found for this review describing chemotherapy patterns for AI/AN patients with TNBC [7]. 

In contemporary breast cancer treatment, genomic expression assay testing plays an important role in guiding chemotherapy decisions. A 2020 SEER study examined the use of the 21-gene recurrence score (RS) for women with hormone positive breast cancers diagnosed 2004–2015. Of the 363,387 women in the cohort, there were 1951 AI/AN women. The study found that AI/AN women were less likely to undergo RS testing (21%) compared to NHWs (22%), representing a small but statistically significant difference [33]. AI/AN women who completed RS testing had a significantly greater likelihood of having high risk RS (18.2% AI/AN compared to 14.3% of NHW) [33]. Among women who underwent RS testing, there were no differences in receipt of chemotherapy between groups. However, among women who were not tested, AI/AN women were more likely than NHW women to receive adjuvant chemotherapy (44% vs. 34%), suggesting that omission of RS testing in AI/AN women might be leading to their overtreatment with chemotherapy [33]. The authors concluded that avoiding unnecessary, ineffective treatment should be a goal for all women, and genomic evidence exists that AI/AN women have more aggressive tumors.

Hormone receptor and Her2/neu receptor status testing is performed at notably lower rates for AI/AN women compared to other groups, which inevitably compels disparities in treatment from the outset forward since receptor status is the nexus of therapeutic decision-making [37]. Genomics is the next horizon for individualized care and improved outcomes. A study on the actionability of molecular targets in breast cancer patients included genomic reports of 1361 breast cancer patients, and there were only two AI/AN patients in the study [38]. AI/AN women need to have representation in clinical research on parity with other racial groups because the research that decodes genomic differences are a pathway to the precision medicine to appropriately tailor therapy and gain health equity.

## Endocrine Therapy

Anti-hormonal therapy is a key component of the treatment of hormone positive breast cancers and strategy for future oncologic risk reduction. In a 2023 NCDB study, the overall survival of patients with hormone positive breast cancer was significantly worse among AI/AN women compared to NHW women [35]. Interestingly, while AI/AN women are commonly reported to have treatment delays and lower receipt of guideline concordant care generically, this showed no difference in the initiation of adjuvant endocrine therapy (ET) between AI/AN and NHW women [35]. The authors found that AI/AN patients had increased rurality, younger age, larger tumor size, and more lymph node positivity, which might be factors driving the worse outcomes for AI/AN women with hormone positive breast cancer rather than differential initiation of ET [35]. 

Adherence to ET is another factor to consider in assessing breast cancer outcomes. ET encompasses a handful of hormone blocking medications with adverse effects that can interfere with quality of life (QOL) and make it difficult to complete the recommended 5–10 years of adjuvant therapy. Reports vary, but approximately 30–50% of all patients with hormone positive breast cancers complete 5 years of ET successfully, and nonadherence is significantly associated with increased likelihood of recurrence [35, 39]. A study using Northern California Kaiser Permanente records focused on urban AI/AN patients eligible for ET and found that 78% of AI/AN initiated treatment compared to 82.5% of NHW women [39]. They also found AI/AN had the lowest rate of ET adherence at 1 year and 5 year follow-up (70.3% and 50.8%, respectively) and greatest annual decline in adherence [39]. Total underutilization, which they defined as the combination of initiation and adherence, was greatest among AI/AN (70.6% versus 58.7%, respectively) [39]. The generalizability of the study is limited since this was confined to Northern California patients, all with Kaiser insurance, but might provide some insight on the patterns of ET usage for other urban AI/AN populations. It conflicts with the aforementioned NCDB study that observed no difference in ET initiation for AI/AN women compared to other groups.

A less studied relevant topic is whether there is any different metabolism of ET amongst racial groups. A study with 94 patients analyzed cytochrome P450 genetic variation associated with Tamoxifen metabolism in AI/AN patients. Their sequencing identified 54 variants, 7 of which were novel variants identified at low frequency [40]. The study raised more questions than answers, including whether the different bioactivation means that different ET dosing or prescribing patterns might be necessary according to cytochrome P450 variant. It begets the recurrent theme that AI/AN representation in clinical research establishing treatment guidelines needs to be a priority for ensuring therapeutic optimization for AI/AN populations.

## Survivorship

### Longterm Outcomes

As repeatedly described, AI/AN women with breast cancer suffer worse long-term outcomes and the poorest survival compared to all other groups, reporting that has not changed over the last 30 years [33]. The most recent large-scale study (2023) on cancer statistics for AI/AN showed for the first time that overall cancer rates among AI/AN individuals were 2% higher than NHW patients [7]. Though a small difference, this noteworthy figure shifts the decades long narrative that AI/AN patients have lower incidence of cancer. The paradigm prevailing in this report, like the multitude of studies preceding it, was that cancer mortality for AI/AN patients was higher at 18% [7]. The authors reported that breast cancer mortality specifically was 8% higher for AI/AN women compared to NHW women (2015–2019) and that rates remained stable in contrast to a decreasing trend for NHW women during this time period.

Cancer statistics for AI/AN derived from IHS repositories has the advantage of avoiding racial misclassification, but disadvantage of leaving gaps in our understanding of screening, diagnosis, and treatment of breast cancer for urban AI/AN. A second study conducted with Northern California Kaiser Permanente data found that breast cancer mortality was 47% greater for AI/AN women compared to NHW women [3]. This is striking because Kaiser Permanente is an integrated health system where presumably all enrolled patients have equal access to cancer care services. This beckons the conclusion that there are other factors driving the disparity beyond access. The study also shows that for this urban location in California, the state with the highest population of urban AI/AN in the country, the breast cancer mortality at 47% is markedly higher than the 8% higher breast cancer mortality for AI/AN nationally. Regardless of geographic location or comparative population, the resounding finding across studies is AI/AN have the worst breast cancer outcomes of all US groups.

Sparse data report that AI/ANs are less likely to receive long-term surveillance specific to breast cancer [8, 35]. This is problematic since active imaging and exam surveillance is the cornerstone of early detection of breast cancer recurrence. Earlier detection leads to earlier action, which can safeguard better long-term outcomes, and it may be that AI/AN women are missing out on this safety net.

### Quality of Life

Beyond lengthening life, cancer survivors rightfully demand QOL. Interventional research and programming on survivorship seem to abound for the general population, but QOL topics for AI/AN women are not well examined. A team of AI/AN investigators developed a web-based culturally relevant AI/AN cancer survivorship program built from community driven research. It was the first to report survivorship issues specifically for AI/AN breast cancer patients with participation of 244 AI/AN breast cancer survivors. 70% of the participants reported that symptoms including pain, fatigue, weakness, depression, and compromised concentration persisted more than 5 years past treatment [41]. 21.3% of the survivors > 5 years out from diagnosis had unmanaged pain [41]. 60.9% of the participants > 5 years out from diagnosis reported difficulty concentrating every day or a few days a week [41]. The participants < 1 year from diagnosis reported worse QOL while longer term survivors reported more favorably [41]. The study described that while hair loss can be universally traumatizing, for some tribes hair reflects spiritual strength and losing it for some resulted in isolation to avoid being regarded spiritually weak [41]. 

The same group conducted another study amongst AI/AN survivors of breast cancer to determine whether barriers during treatment impacted QOL domains in their long-term survivorship. 54% of their participants had difficulty accessing cancer care services, 51.9% reported transportation barriers, and 73.6% cited the lack of cancer care at their local clinics as barriers [42]. It was concluded that the AI/AN survivors’ social QOL was significantly influenced by the barriers experienced when accessing and receiving cancer treatment [42]. AI/AN patient navigation was a solution the authors offered as a resource to address disparities and improve patient satisfaction during treatment and survivorship [42]. The group used their findings to develop interventions hailed as an innovative public health program that over the past 20 years achieved gains in QOL for AI/AN cancer patients, the majority who were AI/AN breast cancer survivors [43]. 

## Discussion

Efforts to improve breast cancer outcomes among AI/AN women are constrained not only by inequities in care delivery, but also by significant limitations in the scope, quality, and frequency of available data. Research focused on AI/AN populations remains disproportionately underfunded and understudied, resulting in an overreliance on outdated or incomplete data sources. For instance, much of the available literature on guideline-concordant care among AI/AN women references a seminal paper now over a decade old [8]. While these foundational studies remain important, they also reflect a persistent data lag—one that impedes the development of timely, evidence-based interventions. Breast cancer incidence and survival data in AI/AN populations are often updated only once every 10–15 years, and in some cases, we continue to cite studies that are 20 years old to address contemporary questions [7]. 

Contributing to these gaps are broader methodological and ethical concerns. AI/AN individuals are frequently misclassified in national cancer registries or grouped into “Other” racial/ethnic categories, further diminishing the visibility of AI/AN cancer disparities [6]. In addition, the limited number of AI/AN-focused cancer researchers—a finite and overextended workforce—has left the field vulnerable to externally driven research agendas that may not align with tribal priorities or cultural values. Research conducted without tribal consent or cultural understanding risks violating principles of AI/AN data sovereignty and undermines trust in academic and clinical partnerships [10]. While there are researchers and institutions working as genuine allies, there are also those who exploit AI/AN data to advance publication metrics without investing in community benefit or long-term relationship building. The consequence is not only missed opportunities for culturally responsive, translatable improvements, but also perpetuation of historical research harms that contribute to ongoing mistrust.

Improving representation of AI/AN communities in clinical trials is both a scientific and ethical imperative. Historically, AI/AN patients have been largely excluded from cancer trials, limiting our understanding of treatment efficacy, toxicity, and survivorship within these populations [44–46]. A growing body of work has shown that successful AI/AN trial recruitment is possible when grounded in culturally appropriate engagement strategies, long-term relationship-building, and community-based participatory research frameworks [45, 47–49]. Establishing what Mainous and colleagues describe as a “circle of trust”—based on transparency, reciprocity, and respect—is essential for trial enrollment, completion, and success [48]. Moreover, trials must address real-world barriers to participation such as geographic isolation, lack of specialty care, transportation challenges, and restrictive eligibility criteria [49]. 

## Conclusions

Disparities for AI/AN women with breast cancer result from multifactorial drivers that include but are not limited to the chronic underfunding of the IHS, workforce shortages, delays in the PRC system, facilities in poor physical and technological condition, geographic isolation, lack of insurance, limited access to multidisciplinary specialty care, policy pitfalls, cultural barriers, the lasting consequences of colonialism, and mistrust. Only by addressing these interrelated factors can we achieve meaningful equity in breast cancer outcomes for AI/AN communities. Approaches should include: expanding access to imaging and specialty services, investing in culturally sensitive outreach and patient navigation, cultural training for providers and healthcare staff that serve this population, improving data quality, and redressing chronic underfunding in tribal and IHS-affiliated healthcare systems. Moving forward, the cancer research community must adopt a more intentional, equity-centered approach to study design and resource allocation. This includes investing in AI/AN researchers, respecting tribal sovereignty, improving race/ethnicity data accuracy, and ensuring that AI/AN patients are not merely participants in research, but co-leaders in shaping its priorities and implementation.

## Key References

- Kratzer, T.B., et al., *Cancer statistics for American Indian and Alaska Native individuals, 2022: Including increasing disparities in early onset colorectal cancer*. CA Cancer J Clin, 2023. 73(2): p. 120-146.
  - ○ Most up-to-date compendium of cancer statistics for AI/AN and first time evidence that cancer incidence is higher for AI/AN populations.
- Javid, S.H., et al., *Guideline-concordant cancer care and survival among American Indian/Alaskan Native patients*. Cancer, 2014. 120(14): p. 2183-90.
  - ○ Seminal paper demonstrating AI/AN cancer patients receive less guideline concordant care, surgery, adjuvant therapy, and surveillance.
- Erdrich, J., et al., *Disparities in Breast-Conserving Therapy for Non-Hispanic American Indian/Alaska Native Women Compared with Non-Hispanic White Women*. Ann Surg Oncol, 2022. 29(2): p. 1019-1030.
  - ○ First study to show AI/AN women undergo higher percentage of mastectomy for early stage breast cancer.
- USCCR, *A quiet crisis: federal funding and unmet needs In Indian country*. 2003, United States Commission on Civil Rights: USCCR.
  - ○ Landmark report revealing disparities in federal funding for AI/AN insufficient to meet basic health needs.
- Nguyen, A.T., et al., *Disparities in Postmastectomy Breast Reconstruction Among American Indian and Alaska Native Women: A Systematic Literature Review and Meta-analysis*. Ann Surg Oncol, 2025. 32(6): p. 4041-4052.
  - ○ A thorough review on reconstruction for AI/AN breast cancer patients.
- Petereit, D.G. and L. Burhansstipanov, *Establishing trusting partnerships for successful recruitment of American Indians to clinical trials*. Cancer Control, 2008. 15(3): p. 260-8.
  - ○ Practical advice and lessons from long-time champions in AI/AN cancer research.
- Mainous, A.G., 3rd, A. Kelliher, and D. Warne, *Recruiting Indigenous Patients Into Clinical Trials: A Circle of Trust*. Ann Fam Med, 2023. 21(1): p. 54- 56.
  - ○ Indigenous conceptual model for AI/AN recruitment from renown Indigenous scholars.

## Data Availability

No datasets were generated or analysed during the current study.
